# Correction: Pandemic Surveillance and Racialized Subpopulations: Mitigating *Vulnerabilities* in COVID-19 Apps

**DOI:** 10.1007/s11673-021-10131-1

**Published:** 2021-10-18

**Authors:** Tereza Hendl, Ryoa Chung, Verina Wild

**Affiliations:** 1 Institute of Ethics, History and Theory of Medicine, Ludwig-Maximilians-University in Munich, Lessingstr. 2, 80336 Munich, Germany; 2 Department of Philosophy, Université de Montréal, C.P. 6128, succ. Centre-Ville, Montréal, Québec H3C 3J7 Canada

**Correction to: Bioethical Inquiry**

10.1007/s11673-020-10034-7

This paper was previously published without the following funding details: This article was supported by the Bundesministerium für Bildung und Forschung (BMBF), grant number FKZ 01GP1791. The original article has been corrected.

